# The use of probiotics in nutrition and herd health management in large Hungarian dairy cattle farms

**DOI:** 10.3389/fvets.2022.957935

**Published:** 2022-09-20

**Authors:** Zsóka Várhidi, Marietta Máté, László Ózsvári

**Affiliations:** Department of Veterinary Forensics and Economics, University of Veterinary Medicine Budapest, Budapest, Hungary

**Keywords:** probiotics, dairy, cattle, nutrition, health management

## Abstract

In the European Union, there is an increasing need for farm animal nutrition products whose positive effects can replace antibiotics that have been heavily used for decades. Thus, the use of probiotics started to increase in the past few years. In this study, a survey on the practical use of probiotics in Hungarian dairy cattle farms and the related experience of farm nutrition experts was conducted. In addition, we surveyed the state of Hungary for probiotics production and distribution. After direct request *via* phone, nutrition experts responsible for farm feeding programs in 23 large commercial dairy cattle farms and eight managers in different feed distributor companies in Hungary filled out the relevant online questionnaires in 2018. The results show that 69.6% of the surveyed farms used probiotics, most often aiming at the optimization of rumen fermentation, protection against stressors, and supplementation of medical treatments. The most common expected beneficial effects of probiotics were more effective calf raising, larger milk yield, more stable rumen fermentation, and improved stress resistance. None of the respondents experienced any negative effects. In Hungary, five out of eight surveyed feed companies produced probiotic products for cattle, and one just distributed them. Company managers generally thought that farm nutrition experts did not have up-to-date knowledge on probiotics, which is why, these products are often not used in an effective way, and the experts' knowledge should be increased. The own experiments of the distributor companies showed that the probiotic products can improve feed digestibility, the efficacy of calf raising, and the reproductive performance of cows. According to the expectations of distributors, the next generation of probiotic products will be microencapsulated and will contain multiple strains and species of bacteria and prebiotics, too. The goal of the product development is to create probiotics with better effectiveness at a reasonable price, having a complex impact and easier application on the herd level. The study showed that probiotics are already frequently used to prevent diseases in Hungarian dairy herds. However, it can be concluded that there is room for improvement, especially concerning the knowledge transfer about the most effective use of probiotic products.

## Introduction

In Hungary, the cattle sector accounted for 10.8% of the total gross output of agriculture in 2020 (1), which is why it is of great importance to sustaining cattle farming. The aggregated value of the sales of veterinary antimicrobial agents in 31 European countries in 2020 was 89.0 mg/population correction unit (PCU), but a large difference was observed between the countries with the highest and lowest sales (range from 2.3 mg/PCU to 393.9 mg/PCU and median value of 51.9 mg/PCU). Hungary had 169.9 mg/PCU sales for food-producing animals, which was well-above the European average (2). Due to the ban on the use of antibiotics for growth promotion and disease prevention at the herd level, and the mandatory reduction in antimicrobial usage, there is a growing demand for products in feed for livestock that will have a similar positive effect on production and replace the antibiotics in the European Union (3, 4). This is one of the reasons why probiotics have become the focus of scientific interest more recently and their use on livestock farms started to increase significantly (5); however, there is no available official or scientific data about the probiotics used in Hungary.

Probiotics have been defined by Food and Agriculture Organization (FAO) and World Health Organization (WHO) as “live microorganisms that, when administered in adequate amounts, confer a health benefit on the host” (6). This definition has been widely accepted by the International Scientific Association for Probiotics and Prebiotics (7). This is applicable to both human and animal nutrition; it does not limit the positive health effects on the digestive tract, does not require the alteration of the gut microbiota, but does require the intake of an appropriate amount (although this amount is not precisely defined) and that the microorganism is in a live state at the time of intake (6). Gram-positive *Bacillus, Enterococcus, Lactobacillus, Lactococcus, Pediococcus*, and *Streptococcus* bacterial strains, as well as *Saccharomyces cerevisiae* and *Kluyveromyces* yeasts, are the most used probiotic agents in feed supplements in the European Union (8, 9). Accordingly, it is advantageous if the species is a member of the normal intestinal flora of the target animal, produces antibacterial substances against potential pathogens, is genetically stable, and can adhere to as well as colonize the intestinal mucosa (8, 10).

According to Szabó and Szabó (11), probiotics reduce the pH of the intestinal contents, produce antibacterial substances, reduce the amount of ammonia and toxic amines, increase the non-specific immune responses, improve feed palatability and carbohydrate digestibility, and synthesize amino acids and vitamins. Changes in the microbiota of the digestive tract also affect the health and productivity of the animals; therefore, rumen fermentation can be manipulated to improve production (e.g., improvement of milk yield and quality, live weight gain, and feed conversion ratio of calves) (12). So far, the most significant positive animal health and production effects of probiotic supplementation in ruminants have been achieved during periods of high stress for the animal and its intestinal flora, i.e., during the periods of weaning, the beginning of lactation, and the change to a feed being rich in easily digestible carbohydrates (5). It is important to consider that probiotics are relatively slow acting, for which they also require the creation of favorable conditions for the reproduction of eubiotic microorganisms, respectively, and therefore they can be used mainly as preventive agents (13).

The aims of the study were to survey (1) the practical use of probiotics in large Hungarian dairy cattle farms including the experience and expectations of farm nutrition experts and (2) the probiotics production and distribution of Hungarian feed distributor companies including the managers' opinion about the possible product developments of bovine probiotics.

## Materials and methods

The surveys were drafted to define the use of probiotics in commercial Holstein-Friesian farms and the views, and future needs of farm nutrition experts as well as the market experience, opportunities, and forecasts of the Hungarian feed distributor companies regarding probiotics. Two different questionnaires were drafted (Supplementary material), which were reviewed by farm nutrition experts (*n* = 3), dairy cattle veterinary practitioners (*n* = 3), academic professionals (*n* = 2), and veterinary and animal science PhD students (*n* = 2) to receive feedback on content. Based on collected feedback, revisions were made before the questionnaires were sent to potential respondents. This survey used a mixed-method approach, which combines the collection of quantitative and qualitative data. The questionnaires contained several open-ended questions that allowed participants to convey their opinion freely. In the first part of this work, data were collected from farm nutrition experts about the number of cows, milk production and reproductive parameters (lactation milk yield, SCC, average lactation number, and calving interval), own feed production, and feed purchase. We also surveyed the practical use of probiotics in the surveyed farms, including the nutritionists' general knowledge, experience, expectations, and future needs on probiotics. In the second part, we gathered data from managers working in different Hungarian feed distributor companies about their probiotic products and the market trends of these product groups as well as the possible product developments and market niches of bovine probiotics.

Commercial Holstein-Friesian farms were included in the first survey based on the following criteria: computerized on-farm records, participation in milk recording, and willingness to provide data to the authors. The questionnaire was available online in Google Forms from 20 October to 1 November 2018. To access the questionnaire, its link was sent to farm nutrition experts, who had access to farm records and were responsible for the farm feeding program, by personal e-mail after a phone call. A total of 23 Hungarian dairy farms were surveyed, and 91.3% of the nutrition experts (*n* = 21), who were employed by the farms and each working on one farm, were agricultural engineers and 8.7% were veterinarians (*n* = 2). Feed production and distribution companies in Hungary were included in the second survey based on the following criteria: distribution and/or production of feedstuffs for ruminants and willingness to provide data to the authors. We also used a questionnaire that was available online in Google Forms from 20 October to 4 November 2018. The managers, who were responsible for feed distribution including probiotics, received the link to access the questionnaire by personal e-mail after a phone call. Responses were received from eight managers working for different feed distribution and production companies.

The participants took part in the survey voluntarily and remained anonymous. Each participant was required to sign a written consent before they began the survey. On average, it took 15–20 mins to fill out the questionnaires. If any questions were raised by the respondents, they were answered and discussed by phone. Each questionnaire has been coded to detect inaccuracies in the data entry. The obtained data were processed in MS Excel (Microsoft Corporation, Redmond, WA, USA).

## Results

### The use of probiotics in dairy farms

A total of 20,738 cows were kept on the 23 farms, which corresponded to 5.26% of the 393,200 Hungarian dairy cow population as of 1 June 2017 according to the official statistical data (14). The smallest surveyed farm had 200 cows, whereas the largest had 2,100, with an average herd size of 901 cows (milking + dry cows), which was higher than the national average of 407 cows. Albeit, all seven regions of Hungary were covered (min. 1 and max. 7 dairy farms per region were involved), it was a non-representative survey. The lactation milk production corrected for 305 days was 10,513 liters on average (*n* = 23; min. 9,000 liters; max. 12,900 liters), out of which 95.8% was marketed (*n* = 23; min. 89.1%; max. 99.5%). The average length of calving interval was 400 days (*n* = 23; min. 375 days; max. 453 days) and the average number of lactations was 2.5 (*n* = 23; min. 1.9; max. 5).

The main feedstuffs on the surveyed farms were maize silage, alfalfa haylage, rye haylage, and meadow hay. The nutrition was the same in both winter and summer on 12 farms (52.2%), while on 11 farms (47.8%), the feeding was slightly modified by season. Namely, in the summer period, easily digestible fiber-rich haylage was given and nutritionists used more feed supplements (e.g., yeast) during heat stress. In the survey, 17 out of 23 dairy cattle farms (73.9%) had their own feed mills and none of them purchased all required forage crops for these mills. There were five farms (29.4%) where forage crops were just grown by themselves and there were 12 farms (70.6%), where forage crops were both purchased and grown (*n* = 17). On the farms with their own feed mills, where forage crops were also purchased and grown, the proportion of purchased crops was, on average, 22% (*n* = 9; min. 10%; max. 40%). Feed supplements were also produced in 35.3% of farms with feed mills, but none of them fully covered their own feed supplement consumption (*n* = 17).

**Figure 1:**
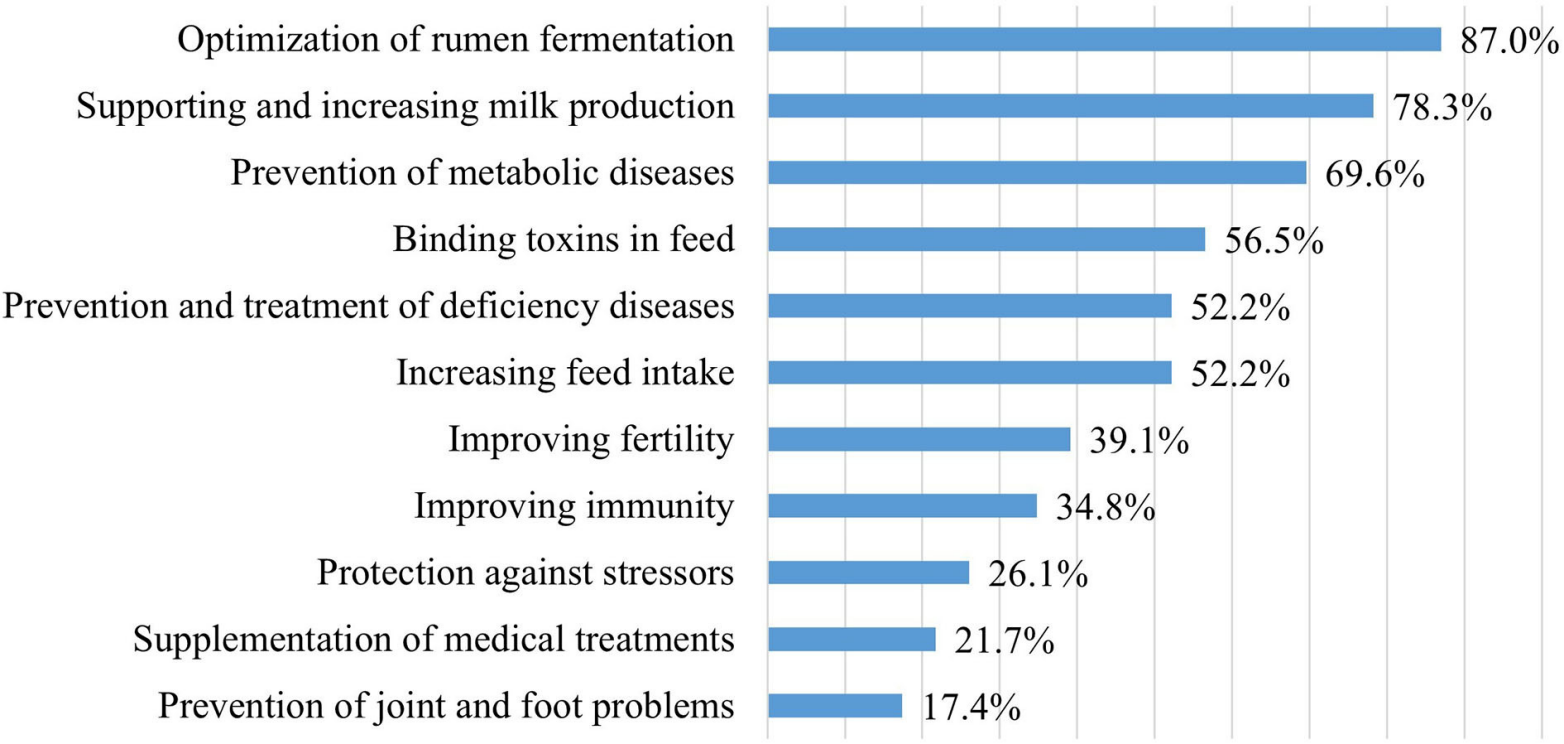
The purpose of the use of feed supplements in dairy cattle farms (*n* = 23).

Feed supplements were used for various purposes in all 23 farms, most often to optimize rumen fermentation, increase milk production, and prevent metabolic diseases (Figure 1).

**Figure 2:**
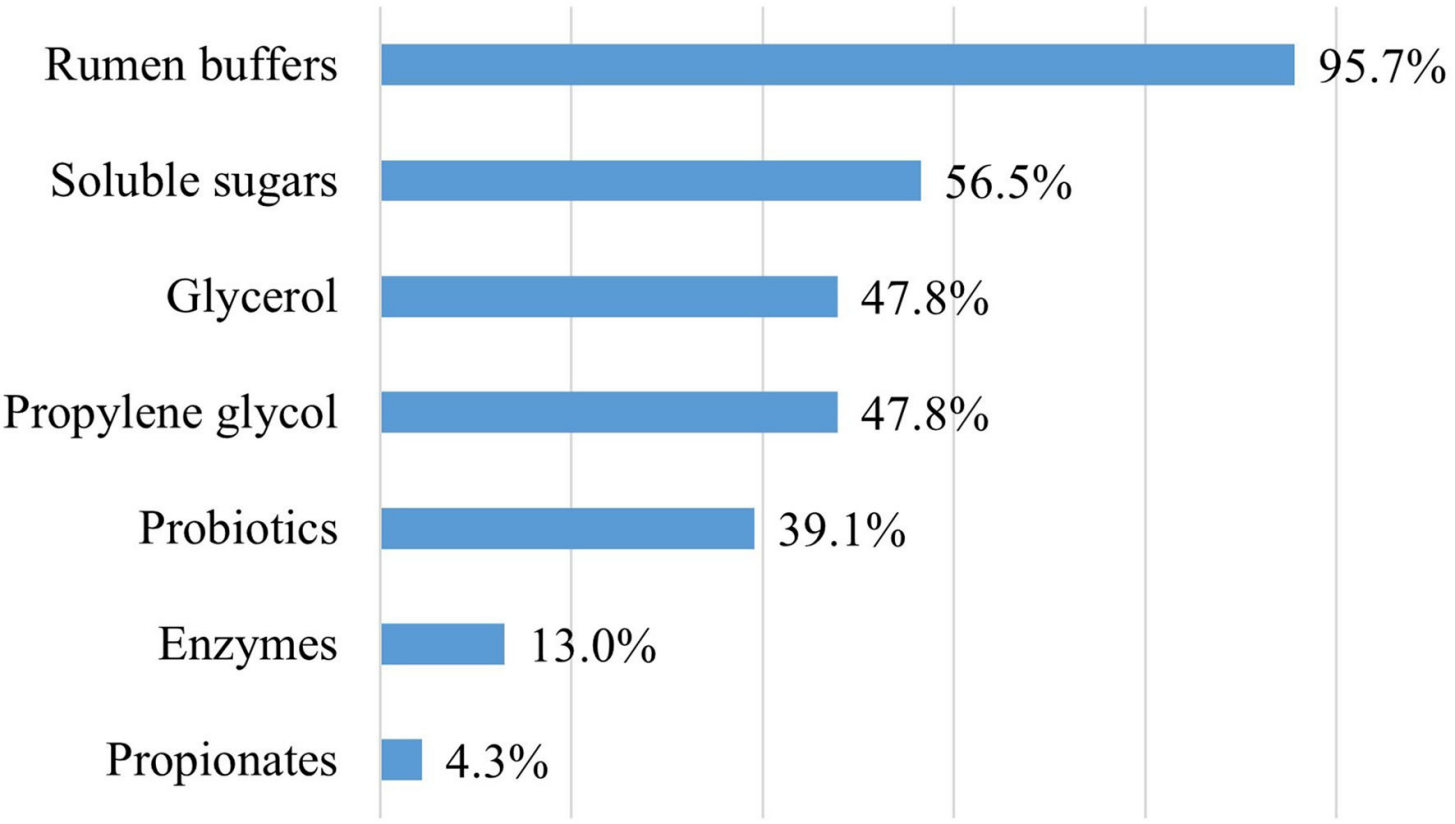
Usage of feed additives to optimize rumen fermentation (*n* = 23). Notes: Propionates, which are organic acids, belong to a functional group of additives, and enzymes belong to the enzyme group in the EU animal nutrition.

Rumen buffers and soluble sugars were used most often to optimize rumen fermentation, while probiotics were used for this purpose in nine farms (39.1%; Figure 2), molasses in three farms (13%), and yeast in two farms (8.7%), respectively.

**Figure 3:**
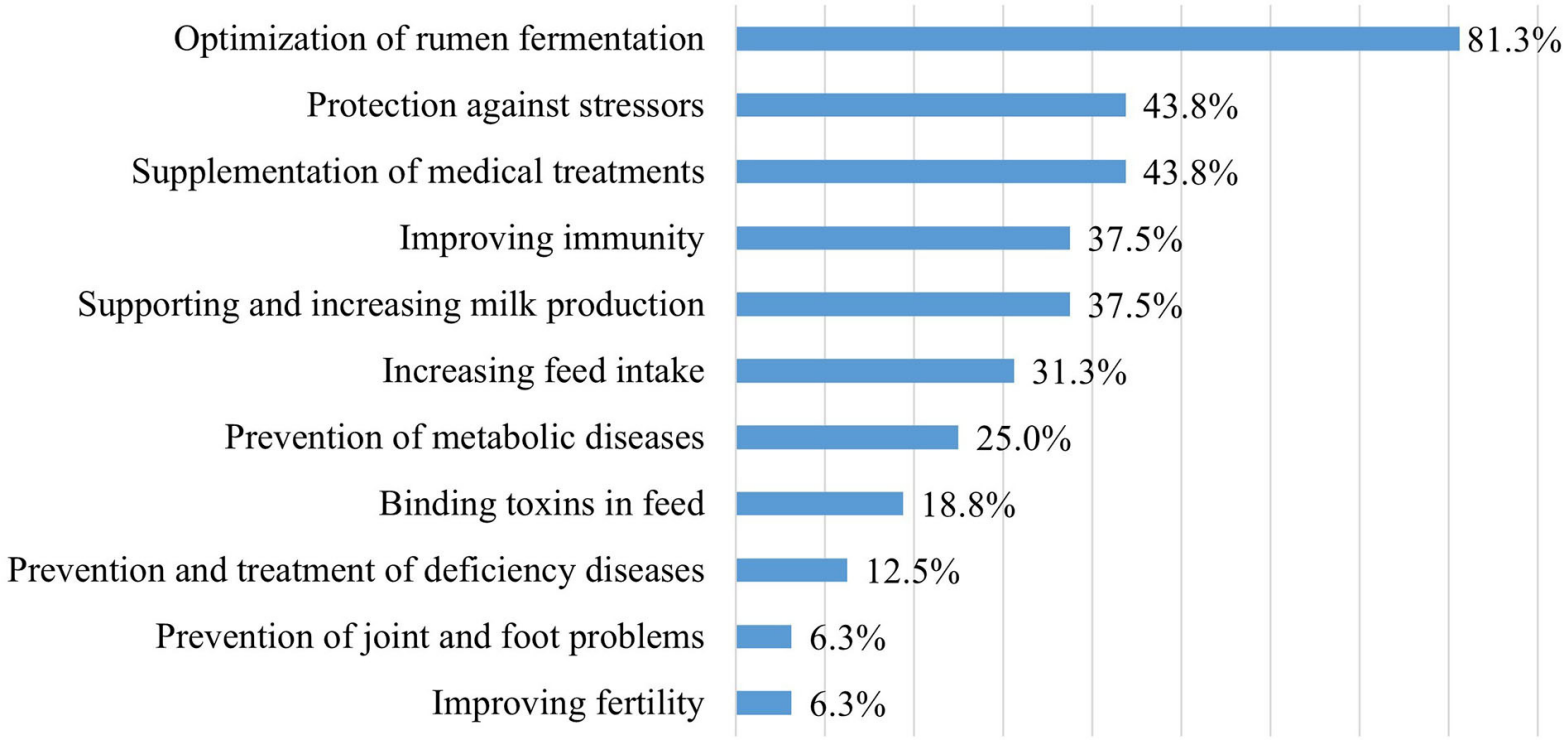
The purpose of using probiotics (*n* = 16).

Of the surveyed 23 farms, 16 used probiotics for some purpose (69.6%), most often to optimize rumen fermentation (Figure 3).

**Figure 4:**
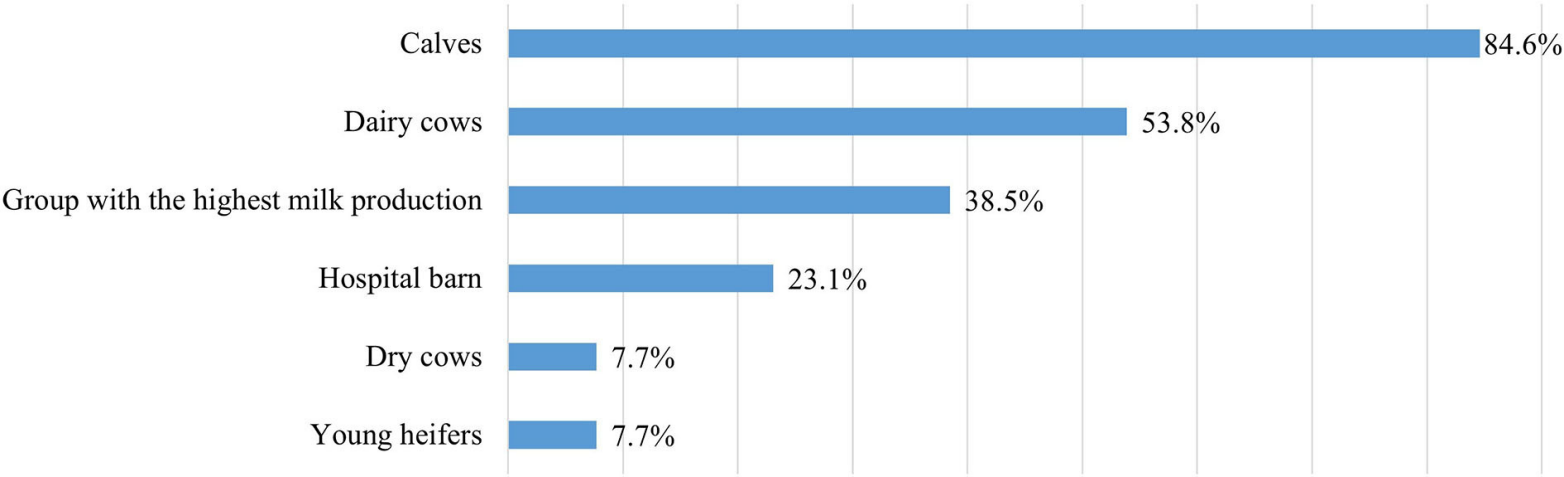
The use of probiotics by animal groups (*n* = 13).

In the farms, probiotics had been used for an average of 6–7 years (*n* = 16; min. 1; max. 20) by using different administration methods at the same time. Probiotics were mixed into the drinking water or administered by drenching in nine farms (56.3%), were mixed into feed in also nine farms (56.3%), were given as powder in seven farms (43.8%), and as a bolus in four farms (25.0%). One respondent mentioned their use in the form of a paste (3.1%). In 43.8% of the farms, probiotics were only used at the group level, in 18.8% at the individual level, and in 37.5% at both levels, respectively (*n* = 16). At the animal group level, probiotics were most often used for calves and milking cows (Figure 4).

**Figure 5:**
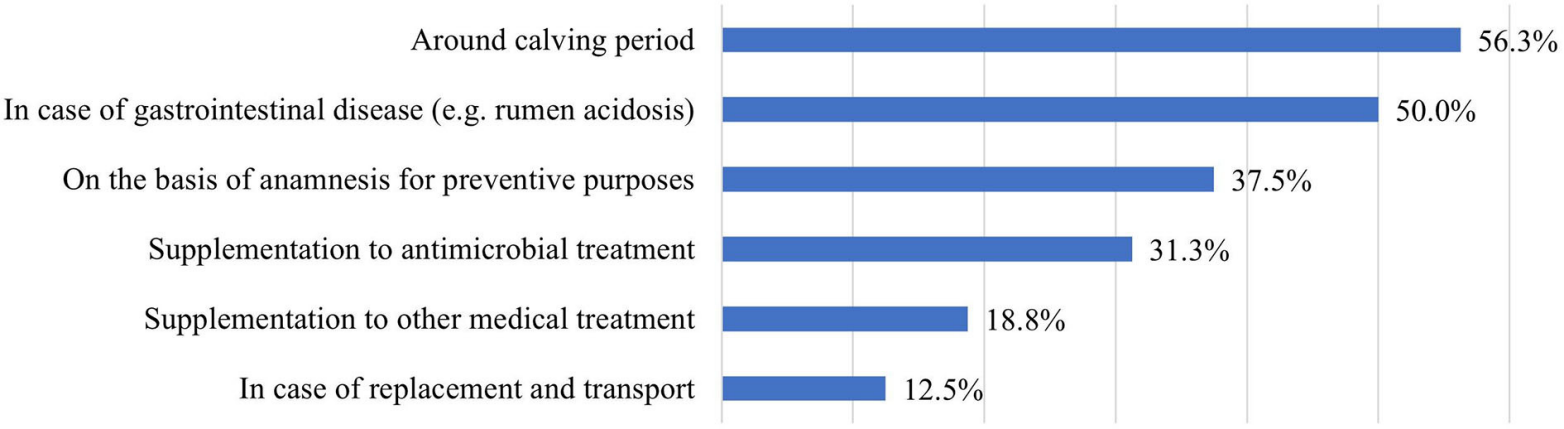
Indications for probiotic feed supplementation (*n* = 16).

Probiotics were most often used on the farms around the calving period or in the case of gastrointestinal diseases (e.g., rumen acidosis; Figure 5).

**Figure 6:**
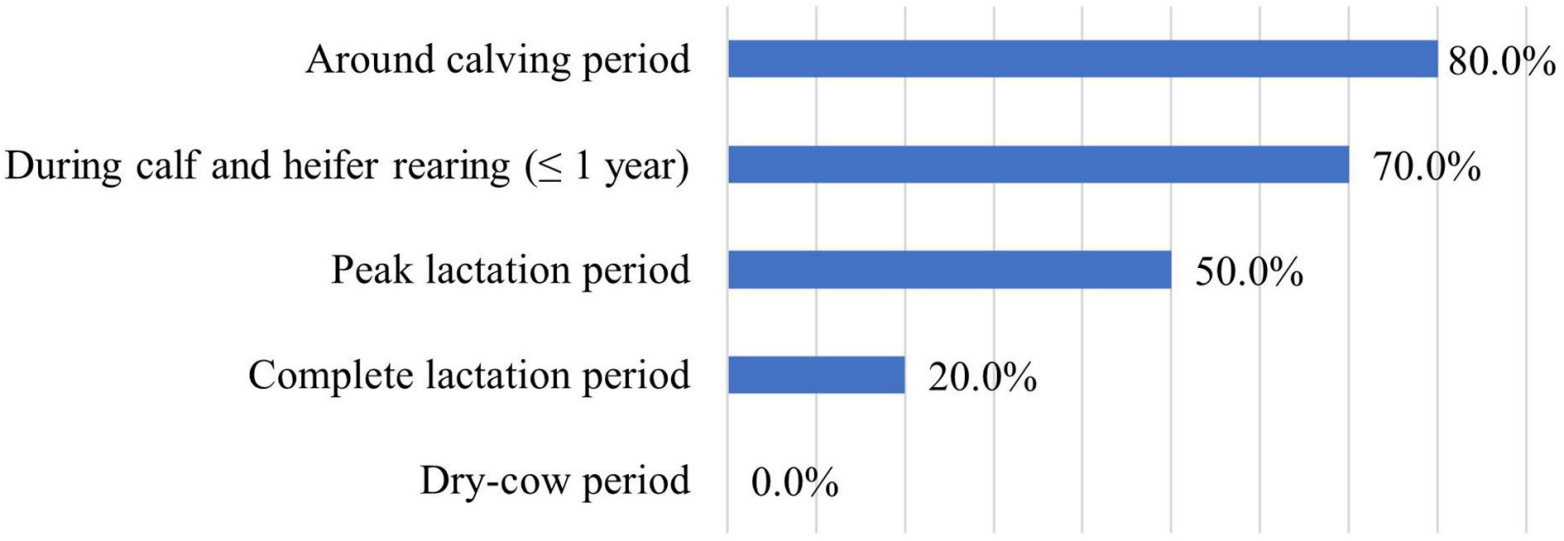
Indications for the periodic usage of probiotics (*n* = 10).

Probiotics were used permanently in 37% and periodically in 63% of the farms. If their use was periodic, the probiotics were most used around the calving period and during calf and heifer rearing, but never used in the dry-cow period (Figure 6).

**Figure 7:**
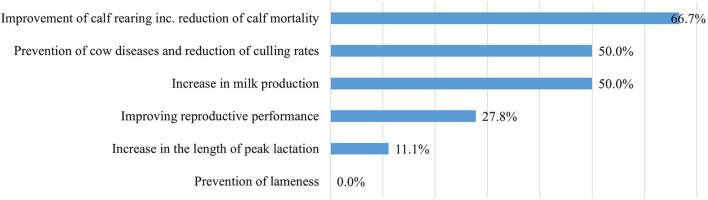
Expected effects of the use of probiotics (*n* = 18).

In two-thirds of the farms (*n* = 12), the probiotics were expected to increase the efficiency of calf rearing and reduce calf mortality. Half of the respondents (*n* = 9) expected the use of probiotics to prevent cow diseases, reduce culling rates, and increase milk production (Figure 7).

**Figure 8:**
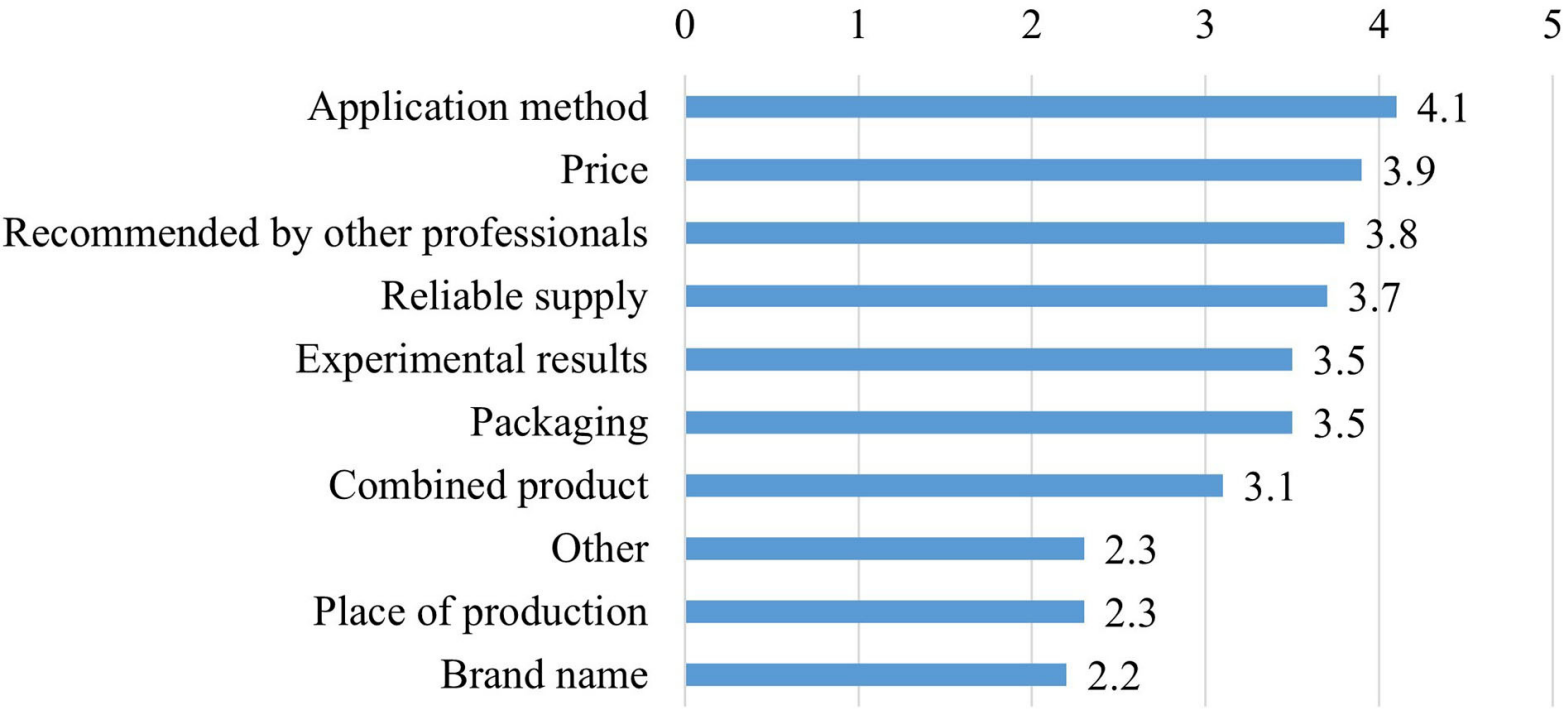
Importance of procurement factors for probiotics (*n* = 19). On a 5-point Likert scale, where 1 = not important at all and 5 = very important; combined products contain more than one probiotic component.

In 30.4% of the farms (*n* = 7), the nutrition experts thought that they had enough information about probiotics and their administration. However, 52.2% of the respondents (*n* = 12) considered the available information to be insufficient and 17.4% (*n* = 4) could not answer this question. The farm experts evaluated the importance of different procurement factors for probiotics on a 5-point Likert scale (1 = not at all important; 5 = very important). Overall, the most important factors were the way of administration, the price, and the experience and recommendations of other professionals, while the least important factors were the place of production and the brand name (Figure 8).

**Figure 9:**
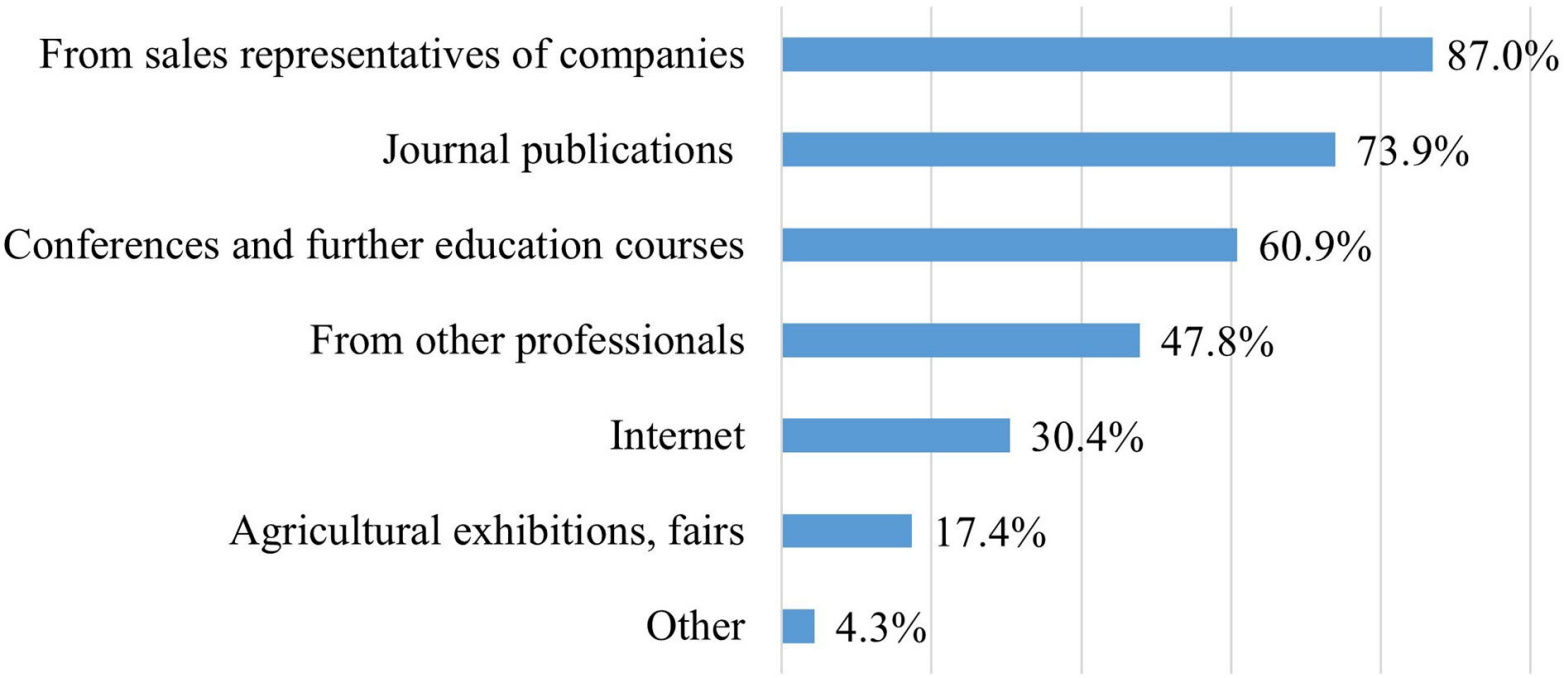
Sources of knowledge of new probiotic products (*n* = 23).

To the question “*How many probiotic products do you know?*,” 30.4% of the farm nutrition experts (*n* = 7) answered that they were aware of more than five products and 60.9% (*n* = 14) answered 3–5 products. However, one expert (4.3%) knew only 1–2 products and another one (4.3%) none. Most farm experts get to know the new probiotic products through sales representatives or from journal publications (Figure 9).

To the open-ended question, “*What do you expect from the new generation of probiotic products?*,” several respondents (*n* = 7) would replace the current probiotic products with more effective, wider spectrum, easy-to-use, and better value-price formulations.

### The production and distribution of probiotics

The surveyed feed distribution companies were founded between 1981 and 2010, and six out of eight (75%) were Hungarian majority owned and two (25%) were international majority owned. In 2017, two companies (25%) had net revenues between 323 and 1,617 thousand EUR (1 EUR = 309.21 HUF), two (25%) had between 1,617 and 3,243 thousand EUR, two (25%) had between 3,243 and 16,170 thousand EUR, and two companies (25%) had revenues over 32,340 thousand EUR. Considering the animal feed market share, two feed distributor companies were in the top 3 in Hungary, one was in the top 4–10 companies, while the other five were not among the top 10 companies in terms of turnover. Four out of eight firms (50%) exported feed to Asia, America, and Europe, primarily to Romania, Moldova, Austria, Slovakia, Russia, Georgia, and Iran. Six out of eight feed distribution companies also had feed production capacities, and all six companies produced feed for both cattle and pigs, five for poultry, four for rabbits, and three for sheep and goats. Two out of eight companies (25%) distributed feed additives only, not ready-made feed. The surveyed companies, that produce compound feed, produced on average between 3,000 and 800,000 tons of complete compound feed per year, of which between 60 and 25,000 tons were produced for cattle. Their main feed supplements for cattle included mycotoxin binders, protected proteins, rumen buffers, polysaccharide enzymes, and yeasts. In Hungary, the total complete compound feed production for food-producing animals was 3.526 million tons in 2017, out of which 350.5 thousand tons were produced for cattle (15).

All the eight surveyed feed companies distributed probiotic-containing products, and they started selling these products between 1988 and 2012. The six surveyed companies with feed production capacities produced between 5 and 130,000 tons of probiotic-containing preparations per year. Five out of these six companies produced probiotic products for cattle, on average 3,112 tons per year, and out of this amount, they produced on average, 3,091 tons per year for dairy cattle. Probiotic products for cattle contained specific live yeast or bacteria (e.g., *Enterococcus faecium*). The income from probiotic products was 27% on average of the total earnings from all feed supplements (*n* = 8; min 5%; max 80%). The income from probiotics for cattle accounted for 33%, on average, of the total income from all feed supplements for cattle (*n* = 7; min 0%; max 72%).

Products available in Hungary during the time of this survey included feed supplements for calves, heifers, and adult cattle as well (mostly targeting peak lactation and heat stress periods). There were different application methods available (predominantly by drenching or mixing into milk replacer), based on the age of the animal, the type of treatment (individual or group level), and other components of the same product. Probiotic components included different bacterial strains (e.g., *Bacillus licheniformis, Bacillus subtilis, Enterococcus faecium*) and/or live yeast (e.g., *Saccharomyces cerevisiae*). Most common additives to probiotics included but were not limited to fructooligosaccharides, vitamins (A, D, E vitamins, biotin), minerals (e.g., manganese, selenium, copper, zinc), L-carnitine, rumen buffers, and enzymes. Some probiotics might contain GMOs. Some distributor companies provided detailed online product descriptions on their websites or in company journals, while others preferred to only provide a list of products or just a general summary of their professional activity and relied more on sales representatives to spread knowledge.

**Figure 10:**
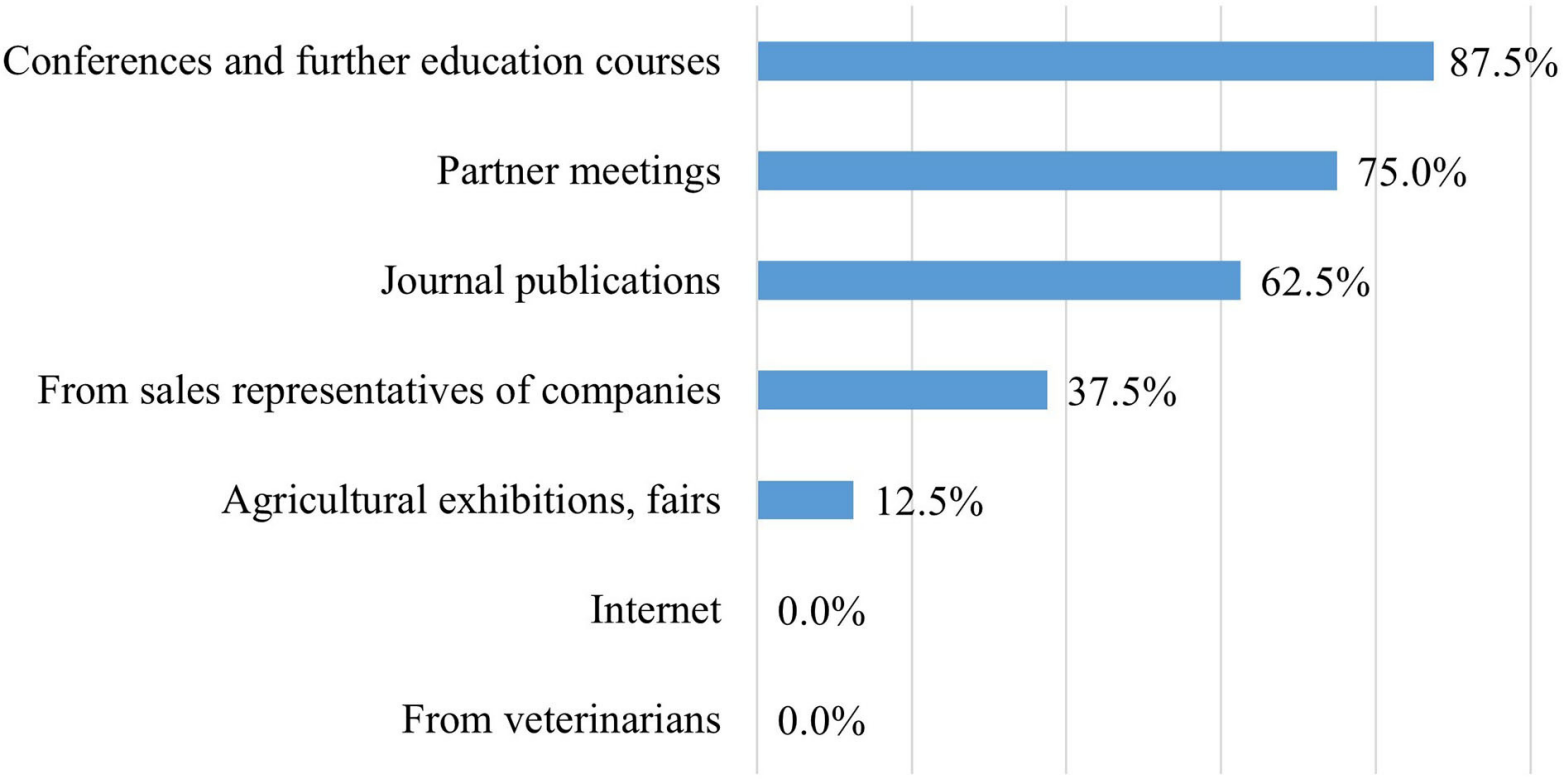
The methods considered most effective by feed distributors to increase the farm nutrition experts' knowledge of probiotics (*n* = 8).

The managers from feed distributor companies were asked to evaluate the farm nutrition experts' awareness of probiotics. Two out of eight company managers (25%) perceived the knowledge of probiotics among the experts responsible for the farm feeding program as good, two (25%) rated it as average, three (37.5%) evaluated it as below average, one could not judge it (12.5%), and none of them perceived it excellent. This corresponds to the fact that more than half of the farm managers are of the opinion that they did not have sufficient information about probiotic products and their applications. According to the feed company managers, the most effective way to increase the farm nutrition experts' knowledge of probiotics could be through conferences, further education courses, or partner meetings (Figure 10). However, most farm experts learn about new probiotic products from sales representatives and journal publications.

**Figure 11:**
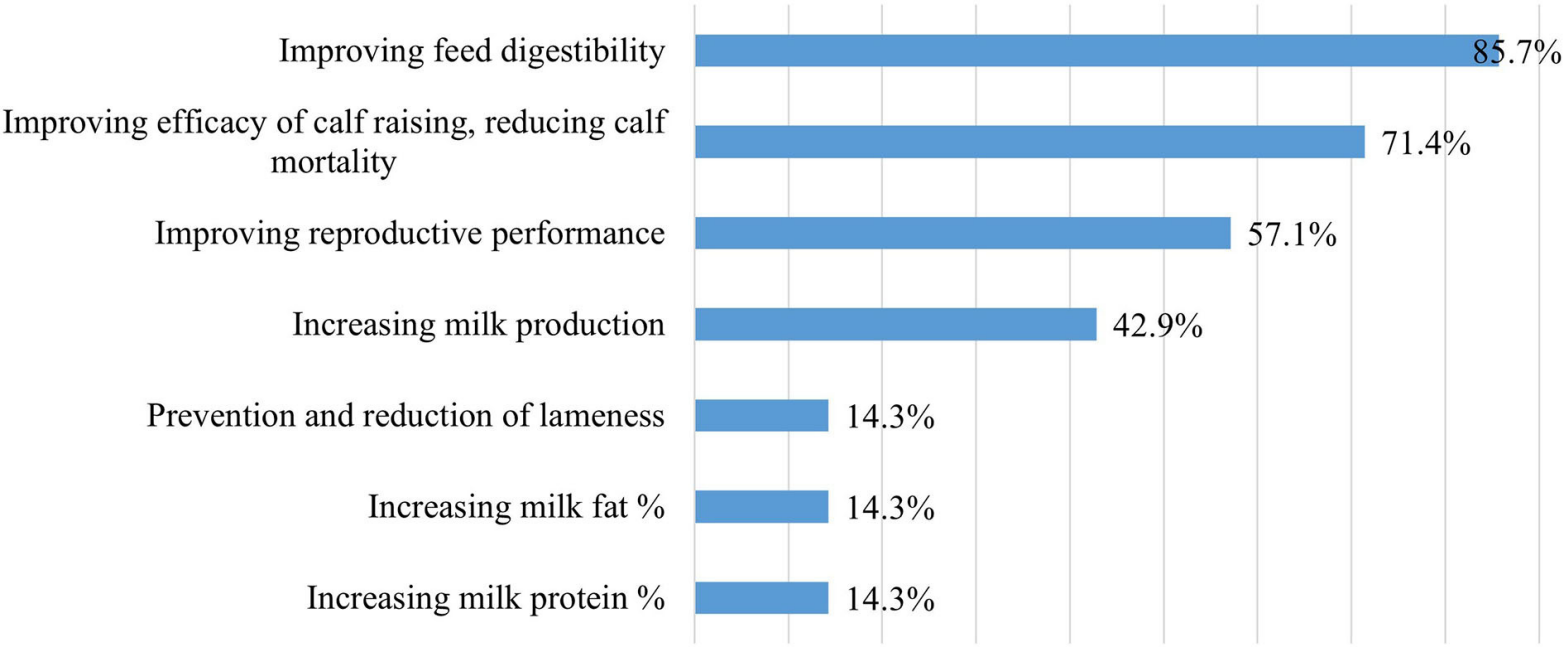
Expected impact of probiotic products in dairy cattle farms based on feed distributor companies' own experiments (*n* = 7).

Based on the own experiments of feed distributor companies, probiotic products were primarily expected to improve feed digestibility, reduce calf mortality, and leverage the cows' reproductive performance (Figure 11). This corresponds to the expected impact of probiotics, as per the opinion of farm nutrition experts, since many of them mentioned the prevention of cow diseases and increased milk production in addition to improving calf rearing efficiency.

According to the experience of feed distributor managers, the most important customer requirements for probiotic products were obvious performance improvements (*n* = 2) and good return on investment (*n* = 2), easy integration into technology (*n* = 1), pathogen control (*n* = 1), reduction of antimicrobial use (*n* = 1), improved feed conversion efficiency (*n* = 1), and reduced rumen acidosis (*n* = 1). After the use of probiotics, the feedback from customers showed a reduction in gastrointestinal diseases (e.g., rumen acidosis, diarrhea; *n* = 3), improved digestion of fiber (*n* = 2), increased milk production (*n* = 1), and less calf mortality (*n* = 1). No negative criticisms were received as regards to probiotic products, but it is important to note that where economic indicators cannot be properly evaluated, financial returns cannot be demonstrated easily, furthermore, the failures of herd health management cannot be avoided with these products.

In the feed distributor managers' opinion, the consumers had different expectations for the next generation of probiotics. These included the development of symbiotic (prebiotic and probiotic) products (*n* = 1), species specificity (*n* = 2), isolation from the digestive tract (*n* = 1), colonization at different parts of the gastrointestinal tract (*n* = 1), ease of use (*n* = 1), improved stability in feed (*n* = 1), and helping to reduce antibiotic usage (*n* = 1). According to the forecast of feed production companies, the next generation of probiotics will contain multiple strains and species of bacteria (*n* = 1), will not contain bacteria carrying antibiotic-resistant genes (*n* = 1), will be microencapsulated (*n* = 1), and will contain both pre- and probiotics (*n* = 1). The next generation of probiotics will reproduce faster in specific areas of the digestive system (*n* = 2) and will bind to the mucous membrane, i.e., they will also have an immune-stimulating effect (*n* = 3) and inhibit inflammatory processes (*n* = 1). Species-specific probiotics with competitive exclusion might be preferred (*n* = 1). Four out of eight companies (50%) did not develop probiotics and two of them (25%) had no local scientific partners, while one company (25%) could rely on the professional support of 1–2 Hungarian research institutes or universities, another one (25%) could have 3–5 domestic scientific partners in the development of probiotics.

The survey respondents' opinion was quite divided on the question of “what the proportion of dairy cattle farms in Hungary was, which did not use probiotic products yet.” One company manager said that around 20% and selecting and evaluating the relevant product was a problem, as opposed to another one who put the figure at around 90%. This share depended on the target group of the products, for example, it was higher for calf nutrition, which is consistent with the fact that both herd managers and companies experienced and expected an improvement in the efficiency of calf raising in relation to the use of probiotics. In their view, in dairy farms that did not use probiotics yet, the application of probiotics could be encouraged by on-farm experiments (*n* = 2) and the distribution of probiotics which could be easily integrated into the farm technology (*n* = 1). In dairy farms, where probiotic products were already used, company managers did not think that the use of probiotics could be increased significantly (*n* = 2), but there would be a demand for better quality probiotic products and their proper application (*n* = 1). They were also convinced that the use of probiotics could be further increased in dairy farms as a part of preventive herd health programs (*n* = 5) and as additional treatments to the unavoidable, curative antibiotic medications (*n* = 7). Other indications for probiotic use might be dysbiosis (*n* = 1), digestive problems (e.g., rumen acidosis; *n* = 3), and lameness (*n* = 1). According to 87% of company managers, efforts to reduce antibiotic use could increase probiotic use. Accordingly, the surveyed feed distribution companies expected average annual growth of 54% in sales of probiotic products in Hungary over a 3-year-long period (*n* = 7; min. 4.5%; max. 300%).

## Discussion

Based on the results of the survey, it can be stated that in the Hungarian dairy cattle farms, probiotic feed supplements were mainly used during calf raising and around the calving period, mostly to increase the efficiency of calf rearing, reduce calf mortality, and optimize rumen fermentation. However, the expected positive effects depend on several factors, such as the microorganisms used as the basis for the probiotic product, the animal-breeding, hygiene conditions on the farm, and the general health status of the animals (13). For instance, under relatively stress-free and temperature-controlled housing conditions, there was no significant difference in the body weight gain and the immunoglobulin levels of Holstein-Friesian calves, which were given milk replacer and starter diet, supplemented with a probiotic product containing *Bacillus* species, compared to the calves in the control group (16).

One of the most common indications of the use of probiotics was to optimize rumen fermentation. An analysis showed that in ruminants, yeast probiotics containing at least one strain of *Saccharomyces cerevisiae* significantly increased rumen short-chain fatty acid concentrations and rumen pH, but the results varied widely. The higher the proportion of neutral detergent fiber in the diet, the better the digestibility of organic matter (17). Yeast supplementation can increase rumen pH and volatile fatty acid concentration while reducing rumen lactic acid concentration (18). Furthermore, several studies reported that yeast-based probiotics in ruminants increased the number of cellulolytic bacteria, which resulted in higher cellulose degradation and microbial protein production (19–21). Pinloche et al. studied the effects of probiotic yeast supplementation in dairy cows at recommended and lower feed intake rates in the early period of lactation (12). Yeast supplementation resulted in higher rumen pH, significantly lower ammonia and lactate concentrations, and significantly higher concentrations of volatile fatty acids, propionate, and butyrate. These values were measurable at both moderate and higher yeast concentrations, but higher yeast concentrations led to better results (12). Probiotics products containing *Bacillus licheniformis* resulted in higher total rumen microorganism content, saturated fatty acid, and propionate concentrations, while the rumen had lower ammonia and lactic acid concentrations (22). According to several studies, probiotics have also been shown to be effective in the prevention or treatment of rumen acidosis. The yeast *Saccharomyces cerevisiae* reduced lactic acid concentrations in the rumen of dairy cows (23), which is likely to have inhibited the development of rumen acidosis (24). In contrast, Hristov et al. reported that *Saccharomyces cerevisiae* had no effect on rumen fermentation. Improvement of feed digestibility in ruminants can also be achieved by using probiotics (25). The use of yeast probiotics increases both fiber digestibility and protein turnover by increasing the number of cellulolytic bacteria in the rumen (19, 26).

Similar to the findings of our survey, several studies showed that certain microorganisms increase milk yield in dairy cows (27–29). Xu et al. investigated the effects of probiotics *Lactobacillus casei Zhang* and *Lactobacillus plantarum P-8* on milk production and milk composition. The use of these probiotic mixtures increased milk yield while reduced somatic cell count by positively affecting the composition of the rumen microbiota (30). Milk yield increased by 2.3 L per cow after daily supplementation with *Enterococcus faecium* (31). Feed supplementation with a combination of *Lactobacillus acidophilus NP51* and *Propionibacterium freudenreichii NP24* resulted in a 7.6% increase in average daily milk yield for Holstein cows (32). Poppy et al. and Maamouri et al. concluded that probiotics containing *Saccharomyces cerevisiae* increased milk production. Using yeasts as feed supplements can increase ruminant dry matter intake and milk production and can improve milk quality (33, 34). Lactic acid-producing bacteria significantly increase milk production, milk protein percentage, and non-fat dry matter content of milk, as well as reduce somatic cell count and mastitis severity by stimulating the immune system (18). Probiotic products containing *Bacillus licheniformis* significantly increased both the milk yield and milk protein content (22).

Several studies examined the effects of probiotics on calf growth and health, showing that probiotics improve average daily gain and feed conversion efficiency in calves (35–39). The fact that calves' probiotic supplementation was more widespread than that of cows is in line with the fact that a large proportion of research studies specifically examined the effect of probiotic supplementation on the body weight gain of calves (35). The feed intake and live weight of calves that were fed a starter diet containing *Saccharomyces cerevisiae* yeast culture were larger on days 42 and 56 of life compared to the calves in the control group and even to those that were given *Bacillus* species supplementation (40). Calves raised on probiotic-supplemented milk could be weaned earlier and had higher body weight at the time of weaning (41). Probiotics containing *Saccharomyces cerevisiae* were shown to improve growth rates in dairy heifers (42). Similarly, a strain of bacteria, *Propionibacterium jensenii 702*, increased weight gain in Holstein-Friesian calves by 25% in the pre-weaning period, and 50% in the weaning period (43). Frizzo et al. concluded that the use of lactic acid probiotic bacteria (e.g., *Lactobacillus acidophilus, Lactobacillus plantarum, Enterococcus faecium, Bifidobacterium* species) increased body weight gain and improved feed conversion efficiency in young calves (35). Probiotics increased the rate of weight gain in 1-week-old beef calves during the first 2 weeks of administration. The rate of increase in weight gain during the first 8 weeks was greater in calves that were less expected to be healthy. Probiotic treatment reduced the incidence of diarrhea, which reduced the need for antibiotic treatment and reduced mortality (44). In a survey by Kelsey and Colpoys, weaned calves were fed a probiotic-supplemented diet for 3 weeks. During this time, an improvement in average daily gain was reported in these calves compared to those not treated with probiotics (45). The improvement was attributed to the feed digestibility enhancing benefits of probiotics, which prevent excessive lactate production and normalize rumen fermentation (46). A study on 6-day-old dairy calves showed that *Enterococcus faecium M74* had a positive effect with significant improvements in body weight and daily weight gain over the entire study period of probiotic treatment (62 days). Probiotic treatment also reduced the incidence of diarrhea (39).

Overall, the most common indications of the use of probiotic products in the surveyed farms (e.g., optimizing rumen fermentation, protection against stressors, strengthening immunity) were mostly the same as those described by Chaucheyras-Durand and Durand (5), who highlighted that the most significant beneficial effects could be achieved during periods of stress for the animals and their intestinal flora (e.g., weaning), and the quantity and quality of milk production could also be significantly improved by probiotic supplementation of ruminant feeding. Certain *Lactobacillus* strains, in addition to their role in maintaining the balance of the intestinal flora, also have anti-inflammatory effects, and thus significantly decrease IL-6, IL-8, IL-10, and TNF-α production in the presence of LPS by reducing gene expression (47). Probiotics can also prevent rumen acidosis and relieve its symptoms by stabilizing rumen pH at a higher equilibrium value by reducing ammonia and lactate concentrations and increasing the concentration of volatile fatty acid, propionate, and butyrate (12, 22, 48). Thus, the main objectives of the use of probiotics in dairy cows are to increase milk production and to improve milk quality, feed conversion efficiency, and animal health status (e.g., reducing rumen acidosis), while in beef cows, the major objectives are to improve live weight gain, feed conversion efficiency, animal health status, and reduce pathogen excretion (5, 49).

Despite the seemingly positive production impact of probiotics, most of the surveyed dairy farms used probiotic preparations only intermittently and there was a significant number of farms that did not yet use probiotics at all, which represents a niche market for feed supplement distributor companies. In addition, while group-based use was more prevalent, individual feed supplementation may become more prevalent as precision livestock farming gains ground. In addition to increasing the quantity of probiotics used, emphasis should also be placed on the proper use of probiotic products. As the questionnaire responses showed, although the farm nutrition experts being responsible for the feeding programs were aware of several different probiotic products, they were often not sufficiently informed on how to use them properly.

The role of probiotics in the fight against antibiotic resistance could also be very useful; however, they might have potential adverse effects. Shridhar et al. (50) used a whole genome sequence-based analysis to detect antimicrobial resistance genes and their results showed that *Enterococcus faecium* carries genes that confer resistance to antibiotics, which are widely used in human medicine (including aminoglycosides, macrolides, lincosamides, tetracyclines, and phenicols). Thus, by treating animals with probiotics, the genes could be transferred to pathogenic bacteria and make them resistant to antibiotics that can be passed on to humans. In the future, probiotic preparations may need to be tested for antimicrobial resistance genes before they can be marketed to food animals (50). In addition, *Bacillus cereus* also produces enterotoxins and emetic toxins (9). Probiotics might also be responsible for systemic infections, adverse metabolic activities, excessive immune stimulation, and gene transfer in the host due to the production of harmful substances by probiotic microorganisms (51). Therefore, there is an urgent need to molecularly investigate the long-term (5–10 years) effects of probiotic microorganisms on the gastrointestinal mucosa (6).

Our results showed that for the probiotic producers and distributors in Hungary, the goal in the product development of probiotic feed supplements is to create more effective, easy to use on herd level probiotics with a wider indication spectrum and better value-price ratio, than the available products in the market. Thus, there was a need for more complex feed supplements, which, for instance, contain both prebiotics and probiotics. The supplementation of feed with fermented wheat germ extract as a prebiotic for suckling dairy calves resulted in a significant reduction in the incidence of respiratory, gastrointestinal, and other diseases, as well as in the use of antimicrobials, and caused an improvement in body weight gain (52). The fermented wheat germ extract supplementation for beef calves brought about a significantly higher live weight gain and lower morbidity and calf mortality rate, and finally, a reduction in the use of antibiotics (52). However, Heinrichs et al. (53) found no significant improvement in calf health (diarrhea, respiratory diseases, general health status) in the prebiotic-supplemented group of calves, but the control group had two times as many calves with diarrhea, and their feed intake was also significantly reduced. The intestinal flora did not differ largely, but the calves in the control group had slightly more *Enterobacter* species, while those in the prebiotic-supplemented group had more *Lactobacillus* species.

According to the questioned farm nutritional experts and probiotic distributor managers, as the use of antibiotics is restricted and must be reduced, probiotics could be brought to the fore as part of the preventive herd health programs instead of the widely used antimicrobial metaphylactic treatments and could more often be complements to the necessary, curative antibiotic treatments. However, the gut microbiota is complex, and it is not yet fully understood how the effects of bacteria benefit the host. Active research is ongoing on the effects of probiotics on live bacteria. Probiotic bacteria have a positive effect on digestive tract function in ruminants by benefiting the microflora and suppressing known gut and food-borne pathogens. But their efficacy and mechanism of action need further investigation (54). Based on the respondents' opinion, the most effective ways to share the newest knowledge about probiotics with farm nutritional experts are the different personal meetings.

According to our knowledge, this was the first scientific study assessing the use of probiotics in nutrition and herd health management in large Hungarian dairy cattle farms, but the limitation of the survey is the non-representative nature of the sample.

## Data availability statement

The datasets generated for this study are available on request to the corresponding author.

## Ethics statement

The revised survey was reviewed by the Scientific Research Committee of the Faculty of Veterinary Science, Budapest and found exempt from human subject protection regulations. The participants provided their written informed consent to participate in this survey.

## Author contributions

LÓ and ZV conceived and designed the study. ZV collected and analyzed the data. LÓ, MM, and ZV contributed to the conceptualization and writing the article. LÓ acquired funding. All authors contributed to manuscript revision, read, and approved the submitted version.

## Funding

The Project was supported by the European Union and co-financed by the European Social Fund: (1) EFOP-3.6.1-16-2016-00024 Innovations for Intelligent Specialization on the University of Veterinary Science and the Faculty of Agricultural and Food Sciences of the Széchenyi István University Cooperation and (2) EFOP-3.6.3-VEKOP-16-2017-00005 Strengthening the scientific replacement by supporting the academic workshops and programs of students, developing a mentoring process.

## Conflict of interest

The authors declare that the research was conducted in the absence of any commercial or financial relationships that could be construed as a potential conflict of interest.

## Publisher's note

All claims expressed in this article are solely those of the authors and do not necessarily represent those of their affiliated organizations, or those of the publisher, the editors and the reviewers. Any product that may be evaluated in this article, or claim that may be made by its manufacturer, is not guaranteed or endorsed by the publisher.
