# Supplementary material for: The use of probiotics in nutrition and herd health management in large Hungarian dairy cattle farms
Source: Front Vet Sci. 2022 Sep 20;9:957935. doi: 10.3389/fvets.2022.957935 (PMC9530397; doi:10.3389/fvets.2022.957935)
Supplement: Supplementary file 1 [file Data_Sheet_1.pdf]

frontiers

Supplementary material

Questionnaire - Role of probiotics in herd health management

1. In which county is the farm located?

____

2. What is the average number of cows on the farm?

____

3. How high is the average lactation milk production, corrected to 305 days (litres/lactation)?

____

4. How high is the marketed milk ratio (%)?

____

5. How long is the average period between calvings (days)?

____

6. What is the average number of lactations?

____

7. Which type of degree does the farm nutrition expert have?

- agricultural engineer
- veterinarian
- other

8. Does the farm (company) have its own feed mill?

- yes
- no

9. If the answer to the previous question is yes, do you purchase or grow crops?

- purchase
- grow
- both

10. If you both purchase and grow crops, how high is the ratio of purchased crops (%)?

____

11. If the farm has its own feed mill, do you also produce feed supplements there?

- yes
- some
- no

12. What are the main feed types used on the farm (plant species and type of feed, e.g. maize silage)?

____

____

13. Does winter and summer feeding differ on the farm (with special attention to heat stress)?

- no
- yes

14. If there is a difference, what is it?

____

____

15. What are the goals of feed supplement usage on the farm (multiple choice question)?

- Increasing feed intake
- Optimization of rumen fermentation
- Improving fertility
- Supporting and increasing milk production
- Improving immunity
- Prevention of joint and foot problems
- Prevention of metabolic diseases
- Prevention and treatment of deficiency diseases
- Supplementation of medical treatments
- Protection against stressors
- Binding toxins in feed
- We do not use feed supplements on the farm.
- Other: ______________________________________

16. What type of feed supplements are used to optimize rumen fermentation (multiple choice question)?

- Probiotics
- Propylene glycol

- Propionates
- Glycerol
- Soluble sugars
- Enzymes
- Rumen buffers
- We do not use any for this purpose on the farm.
- Other: ___________________________________

17. What are the goals of probiotics usage on the farm (multiple choice question)?

- Increasing feed intake
- Optimization of rumen fermentation
- Improving fertility
- Supporting and increasing milk production
- Improving immunity
- Prevention of joint and foot problems
- Prevention of metabolic diseases
- Prevention and treatment of deficiency diseases
- Supplementation of medical treatments
- Protection against stressors
- Binding toxins in feed
- We do not use probiotics on the farm.
- Other: ___________________________________

18. How long have you been using probiotics on the farm (years)?

____

19. What are the application methods of probiotics used on the farm (multiple choice question)?

- Powder
- Bolus
- Mixed into drinking water or by drench
- Mixed into feed
- We do not use probiotics on the farm.
- Other: ___________________________________

20. Are probiotics used on individual or group level on the farm?

- Group
- Individual
- Both
- None

21. If probiotics are used on a group level, in which groups are they used (multiple choice question)?

- Calves

- Young heifers
- Lactating cows
- Group with the highest milk production
- Dry cows
- Hospital barn
- Other: ___________________________________

22. What are the main cases of probiotic feed supplement usage on the farm (multiple choice question)?

- Around calving period
- On the basis of anamnesis for preventive purposes
- In case of gastrointestinal disease (e.g. rumen acidosis)
- In addition to antimicrobial treatment
- In addition to other medical treatment
- In case of rearrangement and transfer
- We do not use probiotics on the farm.
- Other: ___________________________________

23. Are probiotics used continuously or periodically?

- Continuously
- Periodically
- None of the above

24. If probiotics are used periodically, in which cases (multiple choice question)?

- During calf and heifer rearing
- Around calving period
- Complete lactation period
- Peak lactation period
- Dry cow period
- Other: ___________________________________

25. What are the main expected effects of probiotics on the farm (max. 3 different answers can be selected)?

- Increase in milk production
- Increase in the length of peak lactation
- Improving reproductive performance
- Prevention of lameness
- Improvement of calf rearing inc. reduction of calf mortality
- Prevention of cow diseases and reduction of culling rates
- We do not use probiotics on the farm.
- Other: ___________________________________

26. What are the experienced positive effects of probiotics on the farm?

____

____

27. Were there any negative effects of probiotics experienced on the farm?

____

____

28. Do you believe you have sufficient information about probiotic products and their application?

- Yes
- No
- I cannot answer this question.

29. Rate the importance of different factors in choosing a probiotic product (1 = not at all important; 5 = very important)!

- Price
- Packaging
- Application method
- Combined product
- Place of production
- Reliable supply
- Brand name
- Experimental results
- Recommended by other professionals
- Other: ____________________________________

30. If another factor is also important, what is it?

____

31. How many different probiotic products have you heard of?

- 1-2
- 3-5
- More than 5
- None

32. How do you usually get new information on probiotic products (multiple choice question)?

- From sales representatives of companies
- From other professionals
- Agricultural exhibitions, fairs
- Conferences and further education courses

- Journal publications
- Internet
- Other: ___________________________________

33. What would be a reason for you behind changing an already used probiotic product to a new one?

____

____

34. In your opinion, in what aspect should the next generation of probiotic products be different from the current generation? (List at least 3 differences!)

____

____

Questionnaire - Role of probiotics in herd nutrition

1. When was the feed distribution company founded (year)?

____

2. Is the company Hungarian or international majority-owned?

- Hungarian
- International

3. How high was the net revenue of the company in 2017?

- <100 million HUF (<323 thousand EUR)
- 100 - 500 million HUF (323 – 1,617 thousand EUR)
- 501 million - 1 billion HUF (1,617 – 3,234 thousand EUR)
- 1 - 5 billion HUF (3,234 – 16,170 thousand EUR)
- 5 - 10 billion HUF (16,170 – 32,340 thousand EUR)
- >10 billion HUF (>32,340 thousand EUR)

4. Based on market share, how is your company ranked in Hungary?

- Top 3 feed distributors in Hungary
- Top 4-10 feed distributors in Hungary
- Not in the top 10 feed distributors in Hungary

5. Does the company export feed?

- Yes
- No

6. Which countries are your company's main export partners?

____

7. For which type of livestock does the company produce feed (multiple choice question)?

- Cattle
- Sheep and goat
- Pig
- Poultry
- Rabbit
- Other: __________________________________

8. How many tons of ready-made feed are produced for livestock yearly?

____

9. How many tons of ready-made feed are produced for cattle yearly?

____

10. What are your company's main types of feed supplement products for cattle?

____

11. Does your company produce or distribute probiotic products?

- Yes
- No

12. How long has your company been producing or distributing probiotic products (year)?

____

13. What is the yearly amount of probiotic products produced by your company?

____

14. Out of this amount, how much is produced for cattle?

____

15. Out of this amount, how much is produced for dairy cattle?

____

16. What are your company's main types of probiotic products for cattle?

____

____

17. What percentage of the total income from all feed supplements is generated by probiotic products at your company?

____

18. What percentage of the total income from all feed supplements for cattle is generated by probiotic products for cattle at your company?

____

19. How would you rate the knowledge of farm nutrition experts regarding the application of probiotics?

- Excellent
- Good
- Average

- Below average
- I cannot answer this question.

20. In your opinion, what would be the most effective way of educating farm managers and nutrition experts? Choose the 3 most important ways!

- Sales representatives of companies
- Partner meetings
- Veterinarians
- Agricultural exhibitions, fairs
- Conferences and further education courses
- Journal publications
- Internet
- Other: ___________________________________

21. Based on your company's own experiments, which effects can be expected from probiotic products on dairy cattle farms (multiple choice question)?

- Improving feed digestibility
- Increasing milk production
- Increasing milk protein %
- Increasing milk fat %
- Improving reproductive performance
- Prevention and reduction of lameness
- Improving efficacy of calf raising, reducing calf mortality
- Other: ___________________________________

22. What are some general expectations from partners regarding probiotic products?

____

____

23. Based on feedbacks, what are the experienced positive effects from probiotics?

____

____

24. Have you received any negative criticism regarding probiotics and if so, what was it about?

____

____

25. What are your partners' expectations on the next generation of probiotic products?

____

____

26. What are your company’s expectations on the next generation of probiotic products?

____

____

27. If your company develops probiotics, do you have domestic scientific partners whose expertise you can count on?

- Yes, more than 5
- Yes, 3-5
- Yes, 1-2
- We have none.
- We do not develop such products.

28. Who are your main partners in this area?

____

____

29. What percent of Hungarian dairy cattle farms might not use probiotic products currently in your opinion?

____

30. How could these farms be motivated to start using probiotic products?

____

____

31. On the farms that already use probiotics, how much (%) could the usage of probiotic products be increased?

____

32. In which cases or areas of animal health management could the usage of probiotic products be increased?

____

____

33. In your opinion, could the intention of reducing the usage of antibiotics affect the usage of probiotics?

- Yes
- No
- I cannot decide.

34. How do you expect your company’s average annual growth in sales of probiotic products in Hungary to change (%) over the next 3 years?

____

35. How do you expect your company’s average annual growth in sales of probiotic products internationally to change (%) over the next 3 years?

____
